# Effects of intra-abdominal hypertension on maternal-fetal outcomes in term pregnant women: A systematic review

**DOI:** 10.1371/journal.pone.0280869

**Published:** 2023-06-27

**Authors:** Maria Luisa Arruda Correia, Fernando Maia Peixoto Filho, Saint Clair Gomes Júnior, Maria Virginia Marques Peixoto

**Affiliations:** 1 Department of Applied Research in Women’s Health at IFF/Fiocruz, Rio de Janeiro, Brazil; 2 Department of Fetal Medicine, Researcher at IFF/Fiocruz, Rio de Janeiro, Brazil; 3 Department of Clinical Research, Researcher at IFF/Fiocruz, Rio de Janeiro, Brazil; Mayo Clinic Minnesota, UNITED STATES

## Abstract

**Objective:**

To carry out a systematic review to assess the effects of intra-abdominal hypertension on maternal-fetal outcomes.

**Methods:**

The search was carried out between 28^th^ June to 4^th^ July 2022 on the Biblioteca Virtual em Saúde, Pubmed, Embase, Web of Science, and Cochrane databases. The study was registered in PROSPERO (CRD42020206526). The systematic review was performed according to the guidelines of the Preferred Reporting Items for Systematic Reviews and Meta-Analyses: The PRISMA Statement. To assess the methodological quality and control the risk of bias, New Castle was used.

**Results:**

A total of 6203 articles were found. Of these, 5 met the selection criteria for a full reading. The selected studies included a total of 271 pregnant women, of which 242 underwent elective cesarean section and measurement of intra-abdominal pressure via a bladder catheter. In both pregnant women groups, the lowest intra-abdominal pressure values were found in the supine position with left lateral tilt. Prepartum values in normotensive women with singleton pregnancy (7.3±1.3 to 14.1 ± 1 mmHg) were lower than in gestational hypertensive disorders (12.0±3.3 to 18.3±2.6 mmHg). In postpartum, the values decreased in both groups but were even lower in normotensive women (3.7±0.8 to 9.9 ± 2.6 mmHg vs 8.5 ± 3.6 to 13.6 ± 3.3 mmHg). The same was true for twin pregnancies. The Sequential Organ Failure Assessment index ranged from 0.6 (0.5) to 0.9 (0.7) in both groups of pregnant women. The placental malondialdehyde levels were statistically (p < 0.05) higher in pregnant women with pre-eclampsia (2.52±1.05) than normotensive (1.42±0.54).

**Conclusions:**

Prepartum intra-abdominal pressure values in normotensive women were close or equal to intra-abdominal hypertension and compatible with gestational hypertensive disorders even in the postpartum period. IAP values were consistently lower in supine position with lateral tilt in both groups. Significant correlations were found between prematurity, low birth weight, pregnant women with hypertensive disorders, and increased intra-abdominal pressure. However, there was no significant association of dysfunction in any system in the relationship between intra-abdominal pressure and Sequential Organ Failure Assessment. Despite the higher malondialdehyde values in pregnant women with pre-eclampsia, the findings were inconclusive. Given the observed data on maternal and fetal outcomes, it would be recommended that intra-abdominal pressure measurements be standardized and used as a diagnostic tool during pregnancy.

**Trial registration:**

PROSPERO registration: October 9th, 2020, CRD42020206526.

## Introduction

Intra-abdominal pressure (IAP) is the uniform pressure contained in the abdominal cavity and is recognized as one of the main regulatory factors of homeostasis [1, 2]. According to the Abdominal Compartment Society (WSACS), IAP ≥ 12 mmHg is defined as intra-abdominal hypertension (IAH), being recognized as a pathological state that requires attention, and IAP > 20 mmHg is defined as abdominal compartment syndrome (ACS), which is associated with organ dysfunction/failure [3].

According to some studies, pregnancy generates conditions conducive to chronic elevation of IAP, allowing the development of IAH and ACS [4–8].

The occurrence of IAH during pregnancy depends on the relationship between uterine growth and abdominal compliance dictated by the relationship between intra-abdominal volume and IAP [1, 3]. After the 24th week of gestation, the uterus increases exponentially to the point of occupying 61% of the intra-abdominal volume [2]. At that moment, there is a reduction in the compartment’s adaptive capacity and abdominal compliance [5], which generates an increase in IAP [1, 9].

Some Authors have reported that IAH can cause venous congestion, hypertension, hypoperfusion, and systolic cardiac dysfunction [2, 5, 10–13], and multiple organs dysfunction similar to that seen in pre-eclampsia (PE) [14].

Although some studies [2, 10, 11, 15–18] have observed the effects of IAH on adverse maternal-fetal outcomes, the assessment of IAP during pregnancy is underestimated and neglected [2]. Therefore, the present study aims to conduct a systematic review of the literature to assess the effects of IAH on maternal-fetal outcomes.

## Materials and methods

### Design and guiding question

This was a systematic review of the literature undertaken according to the recommendations of PRISMA (Preferred Reporting Items for Systematic Review and Meta-Analyses) with the objective of answering the following guiding question: What are the effects of intra-abdominal hypertension on maternal-fetal outcomes?

#### Protocol and registration

The review’s protocol was registered at the International Register of Systematic Reviews (PROSPERO) under number CRD42020206526 on October 9, 2020. The last update was on June 13, 2022.

#### Information sources and eligibility criteria

Manuscripts written in Portuguese, English, French, or Spanish on non-laboring normotensive pregnant women aged 18 years and above with gestational hypertension, with elective cesarean section and IAP measured via bladder catheter were included.

Articles that evaluated the association between increased IAP/ACS and the presence of abdominal mass, ascites, sepsis, and trauma were excluded. The case series, meta-analysis, and systematic review designs were not the object of the present review.

#### Search strategy

The search strategy in the selected databases was performed considering the following keywords: abdominal compartment syndrome, intra-abdominal hypertension, cardiovascular, and pregnancy, in a combined way using Boolean operators (S1 Table). The search took place between 28^th^ June to 4^th^ July 2022 and was complemented by references identified in selected articles. Cohort studies were selected regardless of the date they were published.

#### Selection of articles, data collection process, quality assessment, and risk of bias

The articles identified in the databases were initially evaluated for their titles and abstracts and scrutinized with the help of Zotero^®^ to identify duplications. Two reviewers independently evaluated them, according to eligibility/exclusion criteria. Doubts and disagreements were analyzed through consensus meetings. Papers that met the eligibility criteria were read in their entirety.

The selected articles were evaluated for their methodological quality, especially regarding risk of bias control based on the Newcastle-Ottawa scale (2021) [19]. The risk of bias quality assessment of the articles included can be observed in the supplementary material (S2 Table). The Newcastle-Ottawa scale [19], developed to assess the quality of non-randomized studies from three perspectives (selection of study groups, comparability of groups, and determination of exposure or outcome of interest), was used in a total of eight sub-items.

#### Data extraction process and summary measures

A specific tool was developed to extract data from the selected studies, which considered: the author’s name, year of publication, place of study, observation period, objectives, evaluated population, inclusion and exclusion criteria, gestational age (GA), body mass index (BMI), birth weight, Sequential Organ Failure Assessment (SOFA) index, placental malondialdehyde (MAD) levels, and the presence or absence of gestational hypertensive disorders.

#### Evaluated variables

IAH was the independent variable analyzed and defined by the parameters of the Abdominal Compartment Society (WSACS) (IAP ≥12 mmHg and ACS when IAP ≥20 mmHg) [20]. IAP was observed in pregnant women with and without gestational hypertension (GH) and PE, diagnosed according to the ACOG definition [21], which dictates the presence of GH when systolic BP ≥ 140 mm Hg and/or diastolic ≥ 90 mm Hg, at least twice within 4 hours, starting at the 20th week of pregnancy and in women with a previously normal blood pressure condition. PE [21] is a type of gestational hypertensive disorder that occurs most frequently after 20 weeks of gestation or near term, associated with signs and symptoms that may or may not be accompanied by proteinuria.

The dependent variables evaluated were the SOFA index and placental MAD levels. The SOFA index is based on six scores relating to the respiratory, cardiovascular, hepatic, hematological, renal, and neurological systems. Each system has a sub-score from 0 to 4 in which its numerical increase reflects the worsening of organ dysfunction [22]. It should be noted that although the SOFA index was created to assess inpatients with sepsis [23] and acute morbidity from critical illness it has been widely validated as a tool for this purpose in a variety of healthcare settings [22].

The serum level of MAD, a product of lipid peroxidation [24], is a biomarker of oxidative stress. It marks a state of imbalance between reactive oxygen species and detoxification mechanisms [25] and is related to the forming of free radicals.

IAP results were evaluated based on mean values, confidence intervals, and p-value before and after emptying the abdominal cavity in cesarean section.

## Result

### Selection of manuscripts

**Fig 1:**
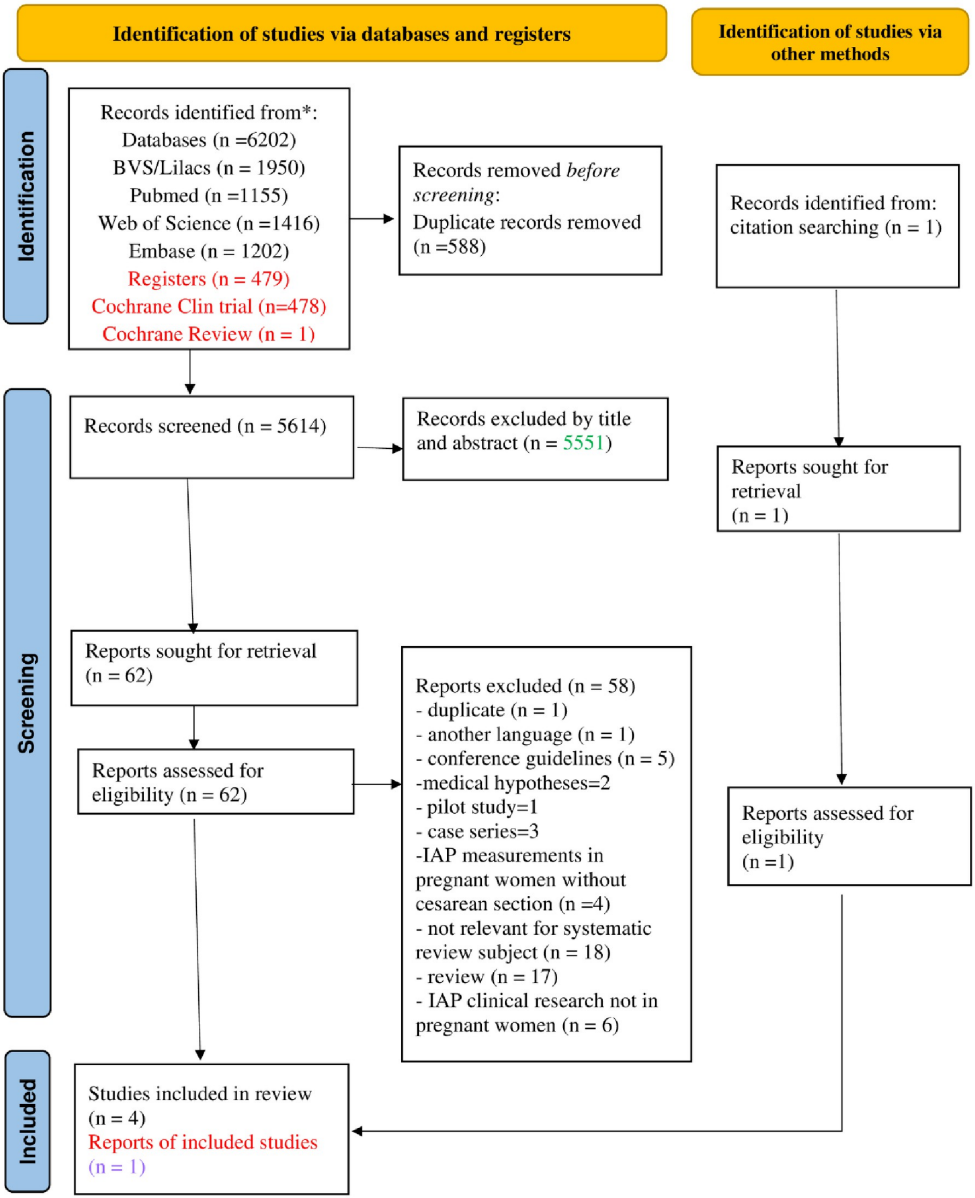
Flow diagram for systematic review which includes searches of databases, registers, and other sources.

Fig 1 contains the flowchart of the number of manuscripts identified in the distinct stages of execution of this systematic review. The search strategy applied in the considered databases returned a total of 6202 studies. To these was added one more article identified in other sources, making a total of 6203. Of these, 588 were removed due to duplicity, and 5551 were excluded after reading the titles and abstracts. A total of 62 articles were read in full, of which five [26–30] were selected.

#### Characteristics of the selected studies

The included manuscripts were published over 12 years, between 2010 and 2022, and the research took place in countries such as the USA [26, 30], India [28, 29], and Turkey [27]. The total number of pregnant women evaluated in developed countries (65) is less than half of those in developing countries (228) (Table 1).

**Table 1:** Characteristics of included studies reporting intra-abdominal pressure measurements in non-laboring pregnant women undergoing elective cesarean section.

First author, year, country	Study				Population			Outcome / comparison group
	Prospective observacional				Non-laboring pregnant women undergoing elective CS			
	Design	Period	Setting	Aim	N	Inclusion criteria	Exclusion criteria	
Abdel-Razeq et al., 2010, EUA	Cohort	April/2007 through April/2008	Obs & Gyn Dep Yale Univ Med School New Haven Hospital	To estabilish normative values of IAP in pospartum women	21	Elective primary or repeat CS	Not schedule CS	IAP after CS with GH/PE and without GH
Ünsal et al., 2017, Turkey	Cohort	Not informed	Obs & Gyn Dep Karadeniz Univ Hospital Trobzon, tertiary referral center	To measure IAP and placental levels of malondialdehyde in patients with PE; To investigate the relationship between IAP and clinical features of PE	70	Study group patients who were diagnosed with hypertensive disorders, PE and HELLP syndrome and control group normotensive pregnant women	Multifetal pregnancies, polyhydramnios, fetal abnormalities, diabetes, thrombophilia, maternal renal disease and severe maternal obesity BMI>40kg/m^2^.	IAP before and after CS in women with and without HG/PE
Arora et al., 2019, India	Cohort	Not informed After consent April /May 2017	Anest & Critical Care Dep Univ and Guru teg Bahadur Hospital New Delhi	To evaluate and compare IAP and its association with organ disfunction/ failure in PE and normotensive patients	58	PE and normotensive patients, singleton pregnancy and spinal anesthesia.	Age < 18 years, polyhydramnios or fetal abnormalities, history of bladder dysfunction or abdominal masses contraindication to spinal block ad post-spinal sensory level lower than T6	IAP before and after CS in women with PE and without PE
Garg et al., 2020, India	Cohort	Not informed After consent October / 2017	Anaest & Critical Care Dep Univ Col Medical Sciences and GTB Hospital, New Delhi	To estabilish and to compare normative values of IAP in supine vs 10º left lateral position; to evaluate the effect on organ disfunction as well as with certain maternal risks characteristics	100	≥37 weeks gestation, elective primary or repeat CS, under single-shot subarachnoid block, including those with complicated pregnancies: PE, diabetes, multiple gestation, previous CS and maternal hemorrhage	Preexisting organ disfuntion/failure and any contraindication to intravesical IAP measurement, such as bladder surgery, hematuria or neurogenic bladder.	IAP before CS in supine and lateral tilt positions by 4 risks factors obesity, PE, multiple gestation previous CS
Narang et al, 2022, USA	Cohort	After consent between August 2020 and December 2021	Obs & Gyn Dep Mayo Clinic College of Medicine	To evaluate the effect of singleton vs twin gestation, as well normotensive vs PE, on IAP during pregnancy	44	≥18 years old, ≥ 28 0/7 weeks of gestational age, non emergent cesarean schedule, with regional anesthesia, including normotensive and PE, diabetes, singleton and multiple gestation	Ruptured membranes, uterine contractions with or without labor, known abdominal-pelvic mass, medical comorbidities that could result in increased IAP, such as preexisting renal disease, neurogenic bladder, pseudotumor cerebri, inflammatory bowel disease, known liver disease, and organ transplant.	IAP before and after CS in left lateral tilt by 4 groups: (1) **Sing**.: N X PE, (2) **Twins**: N X PE, (3) **N**: Sing. X Twins, (4) **PE**: Sing. X Twins. All results compared with maternal BMI.

CS, cesarean section; IAP, intraabdominal pressure; PE, preeclampsia; BMI, body mass index; Sing., Singletons; N, normotensive; GH, gestational hypertension.

The selected articles included a total of 271 pregnant women, divided between 168 normotensives and 103 hypertensives, of which 242 underwent elective cesarean section, with IAP measurement via a bladder catheter.

Only two studies previously performed sample size calculation [28, 29], and bias control in the collection of data was informed in 3 articles. Staelens et al [16] and Garg et al [29] reported that they use a single examiner to avoid intra-observer bias, while Arora et al [28] ensured bias control by blinding the examiner to the clinical picture of the pregnant woman.

With regards to the time of measurement of IAP, one article reported measurement of IAP only before delivery [29], and another after delivery [26], while majority (3 out of 5) reported IAP measurements before and after delivery [27, 28, 30]. Regarding the type of pregnancy, women with a single fetus [26–28] and twins [29, 30] were evaluated.

Two studies sought to standardize IAP measurements in pregnant women by comparing the supine position [26, 29] with the semi-recumbent position [26] or with the 10° left lateral tilt position [29].

#### Assessment of quality and heterogeneity of studies

Assessment of the five selected articles showed a low risk of bias in the studies, all of them being of good quality (S2 Table). Only the study by Arora et al [28] scored 100%, the others having lost points due to measurement bias [26, 29, 30] and single-time follow-up [26, 27]. Given the heterogeneity between the articles regarding the differences in the forms of measurement, it was impossible to carry out a meta-analysis.

#### Different ways of measuring IAP

Observing the means adopted for measuring IAP, it was found that most of the selected articles measured IAP according to the WSACS guidelines [3, 31, 32], that is, zeroing the transducer in the auxiliary midline [27–30] and instilling 25 ml of saline into the bladder [26–29]. The exceptions were the study by Abdel-Razeq et al. [26], which measured by zeroing the transducer in the pubic symphysis, and the study by Narang et al [30], which instilled 50 cc of saline solution. Both used measurement parameters of the Kron technique [33] (Table 2).

**Table 2:** Characteristics of intra-abdominal pressure measurement techniques in the included studies.

Study author, year	N	Measurement time related to CS time		Anesthesia/dermatome	Patient position	Transducer zero reference point	Transducer position	Bladder normal saline injection (mL)
Abdel-Razeq et al., 2010 ^a^	21		within 1h after CS completion	after spinal	supine	pubic symphysis		25
					semirecumbent			25
Ünsal et al., 2017 ^b^	48 ^b1^	at time of admission	24 hous after CS	No dermatone	supine		IAP _MAL_	25
Arora et al., 2019^c^	58	before subarachnoid block and after the onset of sensory block	just after surgery and 2 hours later	sensory block of T6 or higher	supine		IAP _MAL_	25
Garg et al., 2020^d^	100	just before spinal block and prior CS		subarachnoide block	supine and 10° left lateral tilt		IAP _MAL_	25
Narang et al, 2022^e^	44		after spinal block and prior CS	effective up to a T4 dermatome level.	left lateral tilt		IAP _MAL_	50 ^e1^

CS, Cesarean section; IAP, intrabdominal pressure; IAP _MAL_ pressure transducer at the junction of the iliac crest at the mid-axilllary line

^**a**^ Only after CS, measured in both supine and semirecumbent.

^**b**^ IAH when PIA > 15 cm H_2_O

^b1^Only 48 patients with CS with IAP measures

^**c**^ Four IAP measurements

^d^ Two positions supine and 10° left lateral tilt

^e1^ instilled 50cc of saline solution (1cc = 1ml)

Most articles reported factors known to influence the measurement of IAP, such as breathing, vertebral level, or dermatome affected by the anesthetic used and the measurement position [26–30]. The exception in relation to the respiratory factor was Narang et al [30], and in relation to the anesthetized region, the exception was Ünsal et al [27], who measured it after delivery. In addition, Narang et al [30] did not stage the degree of left lateral tilt they used for the measurement.

#### Comparison between pre and postpartum IAP values

In Table 3, we observed that 10.3% (25) of the pregnancies were multiple. Pregnant women with GH/PE accounted for 43% of the sample and were unequally present in the selected articles.

**Table 3:** Comparison of maternal-fetal measurements with IAP values from each study.

author(s), year	Abdel-Razeq et al., 2010 ^a^		Ünsal et al., 2017 ^b^		Arora et al., 2019 ^c^		Garg et al., 2020^d^		Narang et al, 2022^e^	
	without HG	with HG	without HG	with HG	without HG	with HG	without HG	with HG	without HG	with HG
Number of patients	17	4	19	29	29	29	80	20	23^e^°	21^e^_x_
Body mass index	33.3 ± 7.7	37.3 ± 10	24.2 ± 5.7	27.0 ± 7.9	26.7 ± 4.5	27.0 ± 3.5	27.6 ± 3.7^d^_β_	27.6 ± 3.7^d^_β_	31.8±5.2^e1^ 32.6±5.0^e2^	37.5±5.8^e3^ 36.5±9.2^e4^
GWD	37.4 ± 3.0	35.2 ± 5.5	38(mean)	33(mean)	38.9 ± 1.7	37.4 ± 1.9	38.7 (37.7–39.9)^dδ^	38.7 (37.7–39.9)^dδ^	38.9 ± 0.7^e1^ 36.1 ± 1.6^e2^	33.5±2.6^e3^ 33.3 ±1.8^e4^
Birth weight (grams)			3086.3±620.6	1910.9±913.1	2900 (400)	2600 (100)			3331 ± 379^e1^ 4897 ± 890^e2^	2222 ± 1106^e3^* 3688 ± 1111^e4^*
MAD level (μmol/L)			1.42±0.54^b^*	2.52±1.05^b^*						
SOFA index					0.6 (0.5)^c^*	0.9 (0.7)^c^*	^d^* 0.6 ± 0.8 IQR (0–1)			
IAP (mmHg) before CS			13,3 ± 1,9	18,3 ± 2,6	14.1 ± 1^c1^	14.9 ± 0.9 ^c2^	13.1[11.7–14.6]^d1^ 11.7[10.2–13.1]^d3^ 13.7 ± 2.3^d5^ 12.4 (10.2–13.1)^d6^	14.6[14.3–15.8]^d2^ 13.1[12.4–14.6]^d4^ 16.8 (14.6–18.2)^d5^ 14.6 (11.7–16.8) ^d6^	7.3±1.3^e1^ 9.6±2.6^e2^	12.0±3.3^e3^ 12.0 ± 4.4 ^e4^
IAP (mmHg) after CS	5.8 ± 4.6 ^a1^ 10.5 ± 5.5 ^a2^	11.0 ± 9.9 ^a3^ 17.3± 13.6 ^a4^	9,9 ± 2,6	13,6 ± 3,3	9.2 ± 1.2^c3^	10.2 ± 0.8^c4^			3.7±0.8^e1^ 6.2 ± 2.5^e2^	8.5 ± 3.6 ^e3^ 6.5 ± 3.2 ^e4^
P value	0.19 ^a1^ 0.13 ^a2^	0.19 ^a3^ 0.13 ^a4^	<0,05 ^b1^	<0,05^b2^	0,002^c5^	0,001^c6^	< 0,001^d7^ 0,034^d9^ 0,136d^do^	0,001^d8^ 0,034^d9^ 0,136^do^	0.002^e5^ 0.004 ^e6^	

Values are mean ± SD, mean (95%CI) and median [IQR] range- MAD = malondialdehyde, SOFA = Sequential Organ Failure Assessment, CS = caesarean session, GWD = Gestacional week at delivery

The P value presented is relative to the PIA indices

^a^ Only after CS, measured in both ^a1,a3^ = supine ^a2,a4^ = semirecumbent.

^b^ IAP measures in cmH2O- ^b^* p <0.05- ^b1^ pvalue before and after CS in normotensive, ^b2^ pvalue before and after CS in PE

^c^* p = 0.036, ^c^That review choose 2 time measures between 4 from the article data ^c1, c2^ immediately after the onset of sensory block and ^c3, c4^ 2h after surgery before, CS normotensive and PE^c5^, 2hs after normotensive and PE^c6^

^d^_β_ there were 22 obese = IAP supine = (14.9 (14.5–16.5) with determinant—13.1 (11.6–14.6) without determinant, lateral-tilt = 13.3 (12.7–15.0) 11.7 (10.2–12.5)-^dδ^ did not declare the indices separately between normotensive and hypertensive-^d^*(P > 0.05) Supine without PE^d1^ supine with PE^d2^. Lateral tilt without PE^d3^ and lateral tilt with PE ^d4^ -twins in supine position^d5^, twins in lateral tilt^d6^, values of the supine position in relation to the lateral tilt in general^d7^, values of the supine position in relation to the lateral tilt in PE^d8^, ^d9^ values for twins in supine position ^do^ values for twins in lateral tilt position

^e^° = 11 normotensive singleton e 12 normotensive twins; ^e^_x_ = 11 PE singleton, 10 PE twins; ^e1^ = Normotensive singletons, ^e2^ = Normotensive twins, ^e3^ = PE singletons, ^e4^ = PE twins; p value before CS^e5^; p value after CS^e6^. For twins, birth weight is the sum of both infants.*

Based on the WHO classification [34], moderate to severe obesity was detected in all selected studies, with the average sample from developing countries being eutrophic and the one from developed countries obese. BMI values were always higher among women with GH/PE.

During the prepartum period, IAP values were lower in normotensive women with singleton pregnancy (7.3±1.3 to 14.1±1 mmHg) than in women with GH/PE (12.0±3.3 to 18.3±2.6 mmHg). IAP values were, however, found to decrease during the postpartum period in these pregnant women in both groups but the decrease was more in normotensive women (9.9 ± 2.6 to 3.7±0.8 mmHg) compared with women with GH/PE (11.0 ± 9.9 to 8.5 ± 3.6 mmHg). In multiple pregnancies, lower prepartum IAP values were observed in normotensive women (12.4 (10.2–13.1) vs 14.6 (11.7–16.8 mmHg) when compared to women with GH/PE, with a drop in both groups in the postpartum period (6.2 ± 2.5 mmHg vs 6.5 ± 3.2 mmHg).

The IAP measurements in the semi-recumbent position (10.5 ± 5.5 vs. 5.8 ± 4.6 mmHg) were higher than in the supine position in normotensive and GH/PE patients (17.3± 13.6 vs 11.0 ± 9.9 mmHg). A drop in IAP values was also observed among pregnant women with and without complications of single or multiple fetuses in the left lateral tilt position when compared to the supine position.

#### Maternal risk factors and PIA outcomes (SOFA, MAD, prematurity, and birth weight)

The mean GA of all normotensive women with a single fetus was consistent with term delivery (37.4 ± 3.0 and 38.9 ± 1.7 weeks), which was not verified in those with GH/PE (33 and 37.4 ± 1.9 weeks). In multiple pregnancies, GA presented prematurity parameters in both groups (Table 3).

The weight of newborns (NB) was measured in three articles [27, 28, 30]. According to the WHO [35], weight is classified according to gestational age and sex. The weight measured was adequate in most single NBs in normotensive women. However, one [27] woman with GH/PE presented small weight for gestational age. The weight of NBs presented [30] in multiple pregnancies showed measurement bias, hence it could not be classified, as it was presented as the sum of the weights of both NBs.

MAD levels were evaluated in only one study [27] that detected significantly higher placental MAD levels in pregnant women with PE (p < 0.05) than in the control group.

SOFA levels were measured in 40% of the articles in healthy pregnant women outside ICU admission, in order to observe a possible relationship between IAH and organ dysfunction. Both articles observed impairment in the sub-scores of hepatic, respiratory, and hematologic functions and inferred a similar median SOFA index. According to Arora et al [28], although the individual incidence of dysfunctions was clinically high, there was no statistical difference (p<0.05) between the groups of pregnant women. In the analysis of the receiver’s operational characteristics, both studies concluded that there was no significant association between IAP and the occurrence of dysfunction in any of the evaluated systems (P > 0.05).

## Discussion

This systematic review found evidence that prepartum IAP values, both in normotensive pregnant women and in those with GH/PE, were compatible or close to IAH in all measured positions. In the postpartum period, the values are always lower, both in normotensive and in GH/PE women. In 80% of the studies, the IAP values were still at levels close to or equal to the IAH among pregnant women with GH/PE. Studies carried out in normotensive pregnant women and postpartum women under similar conditions also revealed the presence of IAH int the antepartum period and a drop in the postpartum period [15–18].

The importance of controlling IAP during pregnancy was observed in the morbidity and mortality risks associated with IAH. Research indicates that pregnancy would generate a state of chronic IAH that would possibly be related to the development of adverse maternal-fetal conditions [2, 5, 36, 37] and the risk of ACS [38].

The data produced in many studies with pregnant women raise awareness about the severity of the issue. A study involving critically ill pregnant women in the third trimester observed maternal-fetal adverse effects at lower IAP levels than in non-pregnant women with the same pathology [39].

According to another study [40] that evaluated 100 obstetric patients in critical condition, there was an association between the fall in IAP and the prognosis of postpartum survival. In this study, it was observed that all patients who developed IAH were pregnant. However, in 67% of cases, the rates of IAH dropped to < 12 mmHg in the postpartum period, and those who presented with rates ≥ 12 mmHg (33%) died.

Most of the selected articles [27–30] support the current hypothesis that pregnant women tend to have a compensated state of IAH such that failure by some pregnant women to accommodate the content/continent (pregnant uterus/abdominal compartment) relation would lead to the emergence of PE. Narang et al in their study involving twin pregnancies, concluded that the difference in IAP in normotensive women with multiple fetuses was physiological, whereas the increase in IAP among women with PE and singleton fetus was pathological [30].

To verify the possible influence of IAP on maternal-fetal outcomes in pregnant women, other cohorts [10, 11] carried out research with women at various stages of pregnancy. Women who had an increase of more than 4 mmHg within a 2-week period were more prone to complications. When this increase occurred in the early stages of pregnancy, complications were even more severe. Their observations showed that high IAP values between 20 and 24 weeks of gestation preceded the onset of PE.

As for the factors that influence the measurement of IAP, obesity is an important confounding factor to be controlled, as it increases the risk of IAH. Only the study by Narang et al. [30] controlled for preoperative IAP between groups based on the setting of a multivariable linear regression model adjusted for maternal BMI. Garg et al [29] presented single BMI values for both normotensive and HG/PE women but found a different IAP value for the 22 obese patients (BMI>30kg/m^2^) present in their study. Abdel-Razeq et al [26] who had a sample composed only of obese women, however, measured the IAP values only after emptying the uterine cavity, which excluded the influence of uterine weight, but not obesity. The studies, however, pointed out that there was an association between obesity and increased IAP which was more severe among women with GH/PE [26–28, 30].

The measurement body position also affected the IAP values. The search for other measurement positions reflects the question about the standard measurement position in pregnant women. Studies recommend the supine position with left lateral tilt adopted by 40% of the selected papers [29, 30] as the ideal for measurement in pregnant women, as it would avoid compression of the uterus on the bladder [15, 29, 40].

With regards to perinatal outcomes, findings from the selected articles showed that gestational age (GA) was lower among women with GH/PE. In three papers [26, 27, 30], it was compatible with prematurity. Regarding birth weight, one study [27] showed small-for-gestational-age newborns among women with GH/PE, to which the authors associated an influence of IAH. However, both prematurity and small-for-gestational-age newborns were common in women with GH/PE [21, 41], which prevents a more effective causal relationship.

Regarding the weight of multiple pregnancy NBs, the study by Narang et al. [30] made any weight classification unfeasible as the gender was not declared and the weight of each twin NB was not presented separately.

Despite research associating the increase in IAP with impairment of the functioning of internal organs [1, 40], the articles selected for the review [28, 29] did not find a correlation between IAP and SOFA indices in pregnant women. According to studies, it appears that pregnant women at term can tolerate much higher levels of IAP than non-pregnant patients.

Ünsal et al. analyzed the placental levels of MAD in pregnant women and found that the levels were significantly high among patients with IAH [27]. The authors concluded that since most pregnant women who had IAH also had PE, it can not be said that placental ischemia and consequent increase in MAD levels were directly caused by IAH.

The strength of evidence from selected studies was compromised by the small size of most samples. The methodological heterogeneity that included different forms, moments, and measurement positions prevented the execution of a meta-analysis. However, the systematic review was able to collect research that warns about a knowledge gap in relation to pregnancy complicated by IAH and maternal-fetal outcomes.

Research on the subject is still scarce and recent. With this first review on the effects of IAH on pregnant women, we hope to encourage further investigations on the topic, with more robust samples.

## Conclusion

Findings from this study revealed that mean values of IAP in pregnant women at term were close or equal to IAH in normotensive women and compatible with gestational hypertensive disorders even in the postpartum period. A significant correlation was found between prematurity, low birth weight, pregnant women with hypertensive disorders, and high levels of intra-abdominal pressure. IAP values were consistently lower in supine position with lateral tilt in both groups. However, there was no correlation between intra-abdominal pressure (IAP) and Sequential Organ Failure Assessment (SOFA) indices. The levels of malondialdehyde (MAD) were inconclusive.

Given the above, it would be interesting to evaluate the introduction of IAP control during the prenatal period and the creation of a specific measurement for pregnant women to control maternal-fetal effects. However, for a more in-depth assessment of the maternal-fetal risks of increased IAP in pregnant women, a greater number of studies with more studies with larger samples sizes are recommended.

## Supporting information

S1 Table Search strategy. (TIF)

S2 Table Qualitative checklist. (TIF)
