# Supplementary figures and images for: Effects of intra-abdominal hypertension on maternal-fetal outcomes in term pregnant women: A systematic review
Source: PLoS One. 2023 Jun 27;18(6):e0280869. doi: 10.1371/journal.pone.0280869 (PMC10298774; doi:10.1371/journal.pone.0280869)

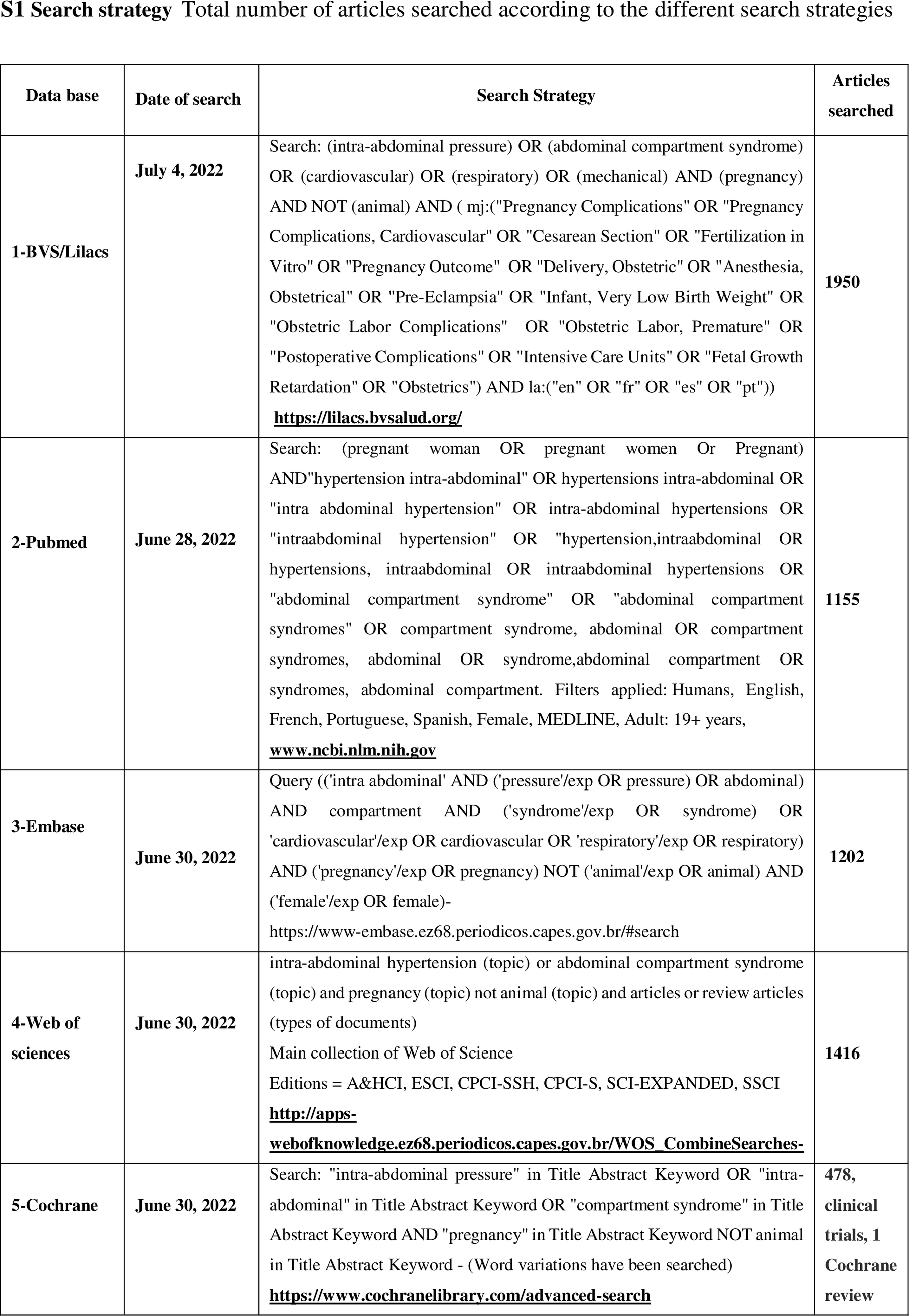

Supplement: S1 Table — (TIF) [file pone.0280869.s001.tif]

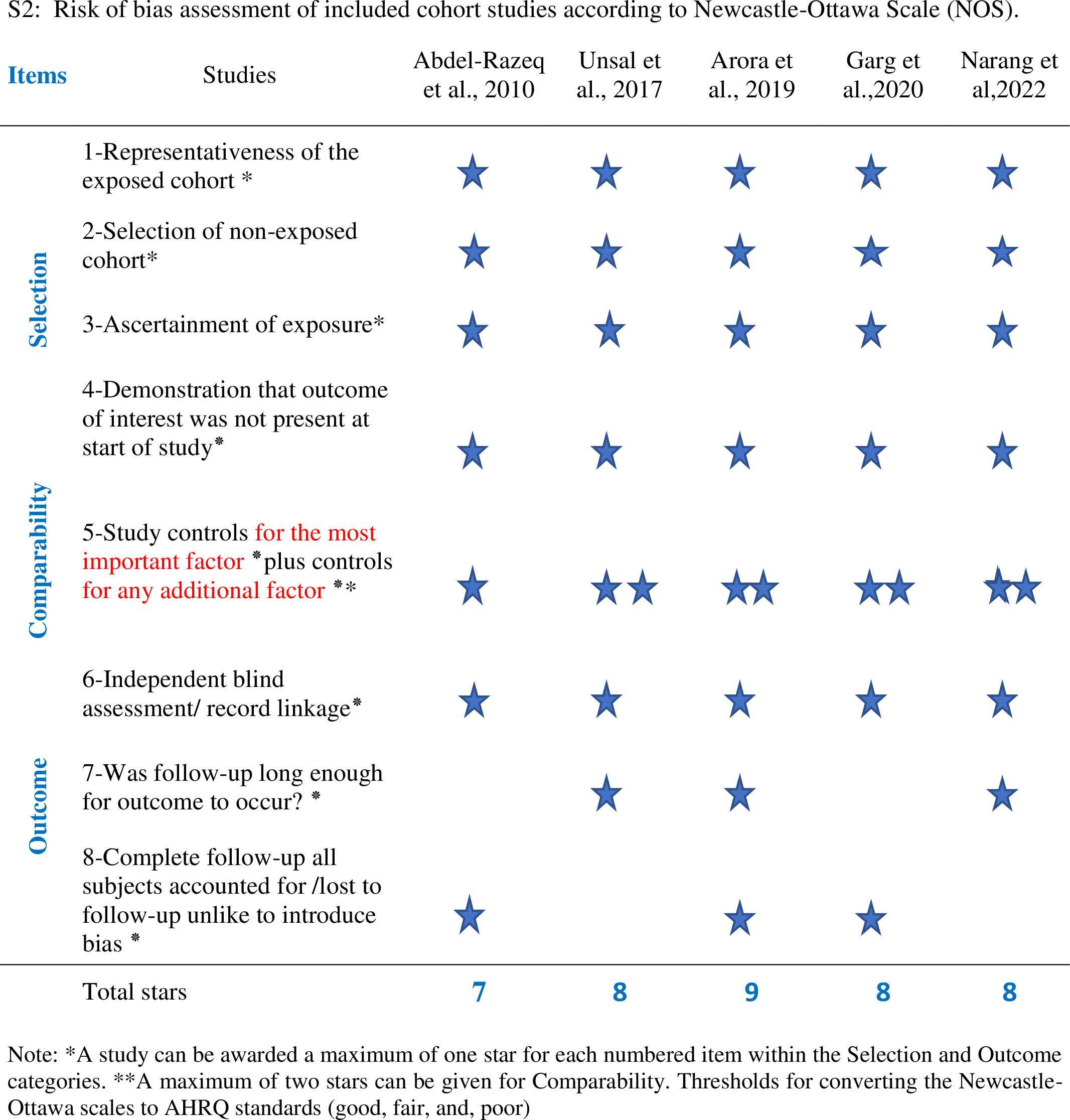

Supplement: S2 Table — (TIF) [file pone.0280869.s002.tif]
